# A Case of Abdominal Sarcoidosis in a Patient with Acute Myeloid Leukemia

**DOI:** 10.1155/2013/379898

**Published:** 2013-02-28

**Authors:** Vadsala Baskaran, Amanda Goodwin, Lavanya Athithan, Ciro Roberto Rinaldi, Alfredo Addeo

**Affiliations:** ^1^Department of Haematology, Pilgrim Hospital, Sibsey Road, Boston, PE21 9QS, Lincolnshire, UK; ^2^Department of Oncology, Pilgrim Hospital, Sibsey Road, Boston, PE21 9QS, Lincolnshire, UK

## Abstract

The allogeneic bone marrow transplantation usually preceded by induction chemotherapy, in fit patients, represents the gold standard in the acute myeloid leukaemia. In the last years, many trials have been set up with the view of improving the number of remissions during the induction by adding new drugs. Several early or late side effects have been described in the literature. We herein present a patient with acute myeloid leukaemia patient who, after chemotherapy, developed ascites that turned out to be abdominal sarcoidosis.

## 1. Case Report

A 68-year-old Caucasian lady presented to the Haematology Department with pancytopenia and palpitations in March 2009. She had a past medical history of uterine fibroids necessitating hysteroannessectomy, diverticulitis, oesophageal reflux, and hypercholesterolemia, as well as a family history of familial *Polyposis coli* for which she was under surveillance. She was taking ranitidine and simvastatin regularly.

Bone marrow biopsy revealed hypercellular bone marrow with architectural distortion of haemopoiesis, a reduction in mature granulocytes and infiltration of myeloblasts (which made up 30–40% of all cells seen within the biopsy) consistent with acute myeloid leukaemia (AML NOS M1 category, cytogenetics 46 XX). She was commenced on the AML16 trial and treated with Daunorubicin, Clofarabine, Gemtuzumab, and nine months maintenance Azacytidine. Allogenic bone marrow transplant was considered but was not pursued at the patient's request. On completion of five months of treatment (October 2010), the patient was asymptomatic and a repeat bone marrow biopsy demonstrated full remission.

**Figure 1:**
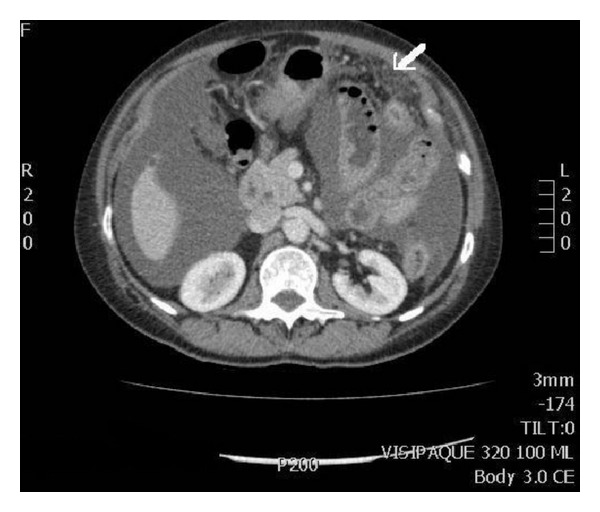
CT scan of the abdomen and pelvis with contrast showing heterogeneous fat/soft-tissue density within the greater omentum (see white arrow). There is also extensive ascites.

Three months later (January 2011), the patient was admitted with abdominal discomfort and distension. On examination, there was evidence of ascites with normal renal and liver function, haemoglobin 12.6, white cell count 3.9, platelet count 316, and an elevated CA125 at 320. Echocardiogram demonstrated good biventricular function. Imaging of the abdomen and pelvis confirmed gross ascites with no focal liver lesion, but there was evidence of fat stranding in the omentum with small omental deposit on the computed tomography of the abdomen and pelvis with contrast (Figure 1). Subsequent MRI showed omental cake and mesenteric deposits raising the possibility of peritoneal neoplasia or mesothelioma. Abdominal paracentesis was performed and 4 litres of straw coloured fluid was drained. The serum-ascites albumin gradient (SAAG) was 1 with LDH 95, white cell count 923 (90% lymphocytes) and no malignant cells seen. Flow cytometry of the ascitic fluid confirmed no evidence of AML. A total body CT scan was performed to rule out any primary solid tumour and there was no evidence of any solid tumour. The patient continued to require frequent ascitic drainage every 3-4 weeks with approximately 4 litres of fluid drained on each occasion.

**Figure 2:**
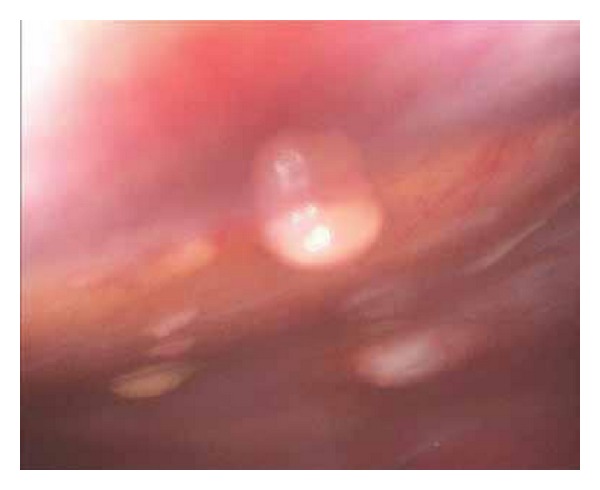
Laparoscopic image of glistening nodules on the peritoneal surface.

A laparoscopic peritoneal biopsy was arranged; macroscopically there was evidence of some miliary deposits on the dome of the bladder and along the peritoneum surface (Figure 2). This was followed by a cystoscopy which did not demonstrate any abnormalities.

**Figure 3:**
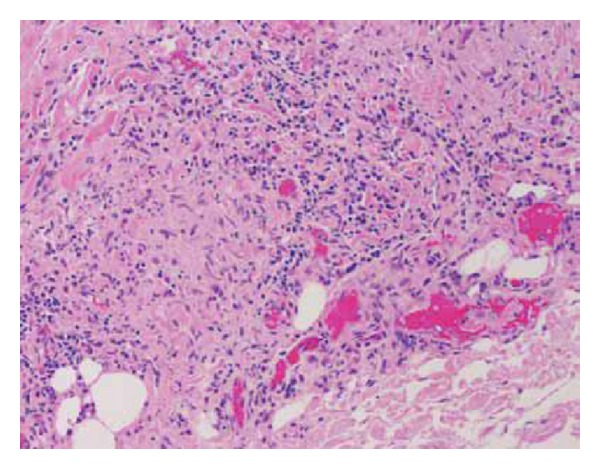
Biopsy of peritoneal nodules showing noncaseating histiocytic granulomas.

Histopathology for the biopsies taken from the peritoneal nodules reported the presence of chronic inflammation and noncaseating granulomata (Figure 3). There were no atypical lymphoid cells or immature granulocytes visualised; no evidence of malignancy or vasculitis and no fungi or acid fast bacilli were grown on extended culture with Grocott and Ziehl-Neelson staining. The patient was admitted 6 times with an interval of 7–10 days to have abdominal paracentesis, every time aspirating 4-5 litres. QuantiFERON test was performed to rule out abdominal tuberculosis and it turned out to be negative.

Based on these results, the patient was commenced on an empirical dose of 25 mg prednisolone and subsequently her symptoms fully resolved. Residual omental thickening was not present in the repeat MRI scan and she no longer required any further paracentesis.

Having excluded the more common causes of granulomata including tuberculosis, fungal infection, autoimmune disease, and neoplasia, this left us with a likely diagnosis of abdominal sarcoidosis. Serum angiotensin-converting enzyme level was normal (54).

## 2. Discussion

Sarcoidosis typically presents with lung involvement or the classic tetrad of fever, bilateral hilar lymphadenopathy, erythema nodosum, and arthralgia; however, 10% of patients present with extrathoracic involvement, most often hepatic [1]. Ascites can occur and is most often transudate, secondary to pulmonary hypertension or portal hypertension due to granulomatous obstruction. However, there are a small number of case reports (a search of the Medline database returned a total of 28) reporting transudative or exudative ascites associated with peritoneal or serosal sarcoid studding [2]. This is associated with a significant elevation in serum CA125, as seen in our patient. Isolated ascites in sarcoidosis is usually benign and highly responsive to steroids, a feature again seen in our patient.

ACE is produced by sarcoid granulomas, and serum ACE levels are used to correlate with disease load [2]. Elevated serum ACE occurs in approximately 60% of patients with sarcoidosis. However, having a normal ACE level as in our patient does not exclude the diagnosis.

There has been several previous case reports of sarcoidosis associated with a diagnosis of AML. Of these, only three reported AML preceding the diagnosis of sarcoidosis, with intervals ranging from 11 months to 17 years [3–5]. The case presented here adds to list, with an interval of 8 months between the diagnosis of AML and sarcoidosis. To our knowledge this is the only case report of peritoneal sarcoidosis following a diagnosis of AML. There have been no previous cases revealed on extensive literature searches.

The exact relationship between AML and sarcoidosis is unclear. It has been hypothesised that the granulomatous inflammation observed in sarcoidosis may occur in reaction to tumour-associated antigens that are widespread in AML. In addition, the transmission of sarcoidosis or sarcoidosis-inducing pathogens, for example, via bone marrow transplantation, has been considered [6]. Our patient did not receive a bone marrow transplant, but she was treated with several chemotherapeutic agents as part of the clinical trial. Granulomatous disease has been associated with exposure to chemotherapy agents, for example, capecitabine, interferon beta, and particularly heavy metals or methotrexate [7, 8]. It is not clear whether this was a factor in the case presented here. There have been no similar cases in the AML16 study cohort to our knowledge, but this has been raised as a potential adverse event with the trial centre. The relationship between AML and sarcoidosis is an area that requires further study.

## 3. Conclusion

The distinctiveness of this case is the uncommon presentation of sarcoidosis, in association with a preexisting diagnosis of AML. As mentioned in the discussion, our patient is a participant of a clinical trial and a possible conjecture is whether one of the chemotherapeutic drugs used could have influenced the development of granulomas in our patient. However no similar symptoms have been reported in other trial patients to our knowledge.

We have highlighted this case as a possible late adverse event to the trial centre and to the competent authority.

## References

[1] Thomas KW, Hunninghake GW (2003). Sarcoidosis. *Journal of the American Medical Association*.

[2] Ebert EC, Kierson M, Hagspiel KD (2008). Gastrointestinal and hepatic manifestations of sarcoidosis. *American Journal of Gastroenterology*.

[3] Ozbudak O, Ozbudak IH, Wang KP (2009). Association between acute myeloblastic leukaemia and sarcoidosis. *West Indian Medical Journal*.

[4] Pagano L, Visani G, Ferrara F (1998). Contemporaneous acute myeloid leukaemia and sarcoidosis. Report of three cases. *Sarcoidosis Vasculitis and Diffuse Lung Disease*.

[5] Isoda M (1996). Cutaneous sarcoid reactions during long term remission in a patient with acute myelogenous leukemia. *Journal of Dermatology*.

[6] Padilla ML, Schilero GJ, Teirstein AS (2002). Donor-acquired sarcoidosis. *Sarcoidosis Vasculitis and Diffuse Lung Diseases*.

[7] Chakravarty SD, Harris ME, Schreiner AM, Crow MK (2012). Sarcoidosis triggered by interferon-Beta treatment of multiple sclerosis: a case report and focused literature review. *Seminars in Arthritis and Rheumatism*.

[8] Kang SM, Baek JY, Hwangbo B, Kim H-Y, Lee G-K, Lee HS (2012). A case of capecitabine-induced sarcoidosis. *Tuberculosis and Respiratory Diseases*.

