# Erratum to “A Case of Abdominal Sarcoidosis in a Patient with Acute Myeloid Leukemia”

**DOI:** 10.1155/2013/240545

**Published:** 2013-05-12

**Authors:** Vadsala Baskaran, Amanda Goodwin, Lavanya Athithan, Alfredo Addeo, Ciro Rinaldi

**Affiliations:** ^1^Department of Haematology, Pilgrim Hospital, Sibsey Road, Boston, Lincolnshire, PE21 9QS, UK; ^2^Department of Oncology, Pilgrim Hospital, Sibsey Road, Boston, Lincolnshire, PE21 9QS, UK

Author names are incorrectly listed as follows:

 Vadsala Baskaran, Amanda Goodwin, Lavanya Athithan, Ciro Roberto Rinaldi, and Alfredo Addeo.

They are now listed as follows:

 Vadsala Baskaran, Amanda Goodwin, Lavanya Athithan, Alfredo Addeo, and Ciro Rinaldi.

